# Short communication: ultrasound-guided percutaneous cryoanalgesia of intercostal nerves for uniportal video-assisted thoracic surgery

**DOI:** 10.1186/s13089-022-00284-4

**Published:** 2022-07-30

**Authors:** Matías Nicolás, Cecilia M. Acosta, Marcelo Martinez Ferro, Agustín Alesandrini, Sofía Sullon, Facundo A. Speroni, Gerardo Tusman

**Affiliations:** 1 Department of Surgery, Hospital Privado de Comunidad, Mar del Plata, Argentina; 2 Department of Anesthesiology, Hospital Privado de Comunidad, 7600, Mar del Plata, Buenos Aires, Argentina; 3 Department of Surgery, Fundación Hospitalaria, Buenos Aires, Argentina

**Keywords:** Ultrasound, Cryoanalgesia, Twinkling artifact, Thoracic surgery, Pain

## Abstract

**Background:**

Pain after thoracic surgery impairs lung function and increases the rate of postoperative pulmonary complications. Ultrasound-guided percutaneous cryoanalgesia of intercostal nerves constitutes a valid option for adequate postoperative analgesia. A key issue for a successful cryoanalgesia is placing the cryoprobe tip close to the intercostal nerve. This report describes an ultrasound technique using a high-resolution ultrasound probe to accomplish this goal.

**Findings:**

Images of five anesthetized patients undergoing uniportal video-thoracoscopic surgeries are used as clinical examples. In the lateral position, a high-frequency 12 MHz probe is placed longitudinally at 5–7 cm parallel to the spine at the 4th, 5th, and 6th ipsilateral intercostal spaces. Ultrasound images detect the intercostal neurovascular bundle and a 14G angiocath is placed beside the nerve. The cryoprobe is inserted throughout the 14G catheter and the cryoanalgesia cycle is performed for 3 min. Two ultrasound signs confirm the right cryoprobe position close to the nerve: one is a color Doppler twinkling artifact that is seen as the quick shift of colors that delineates the cryoprobe contour. The other is a spherical hypoechoic image caused by the ice ball formed at the cryoprobe tip.

**Conclusions:**

Ultrasound images obtained with a high-frequency probe allow precise location of the cryoprobe tip close to the intercostal nerve for cold axonotmesis.

**Supplementary Information:**

The online version contains supplementary material available at 10.1186/s13089-022-00284-4.

## Background

Thoracic surgeries, including minimally invasive uniportal video-assisted thoracic surgery (u-VATS), are painful procedures that have an impact on patient outcomes and hospital costs [1, 2]. Regional anesthesia techniques infusing local anesthetics allowed proper analgesia despite these therapies being limited to a few postoperative days, and with the risk of local infection and catheter dislodgment [2, 3]. Cryoanalgesia is a good option for pain treatment after thoracic surgery [4]. Cold applied directly on intercostal nerve surface induces axonotmesis with transitory and reversible axonal disruption, which develops weeks to months of complete dermatome analgesia [4–6]. Cryoanalgesia shows good analgesia after thoracic surgeries [7, 8]. However, half of the published studies failed to demonstrate better analgesia quality over standard analgesic techniques [9]. Limited performance of cryoanalgesia can be related to factors that lead to suboptimal intercostal nerve axonotmesis, comprising target temperature, size of the ice ball formed at the cryoprobe tip, rate of freezing and thawing, and duration of the freezing cycle [4, 6, 10]. Another important factor is the proximity reached between the cryoprobe ice ball and the intercostal nerve, which would be as close as possible to get proper neuropraxia [10]. The lack of neurolocation with the blind placement of the cryoprobe below the upper rib could increase the rate of inadequate analgesia. This crucial point is highlighted by Hardy, who showed in cadavers that only 17% of the intercostal nerves lie in the subcostal groove [11].

Percutaneous cryoanalgesia guided by ultrasound (US) images has been recently described in a few publications [10, 12–15]. These reports used a convex 3–5 MHz US probe with low-resolution images that could not identify the tiny intercostal neurovascular bundle. Thus, a convex transducer guided the cryoprobe into the subcostal groove of the upper rib, assuming that the intercostal nerve has this anatomical disposition [13–15]. Taking into account the variability of intercostal nerve position, Djebbar et al. addressed the importance of neurolocation using high-resolution US images for optimal cryoanalgesia [10].

In this short communication, a US-guided percutaneous cryoanalgesia technique using a high-frequency 12 MHz probe is presented. We describe US signs that identify with more accuracy the intercostal neurovascular bundle, the cryoprobe tip, and the pleural surface to assure adequate and safe cryoanalgesia for u-VATS.

## Percutaneous cryoanalgesia technique

**Fig. 1:**
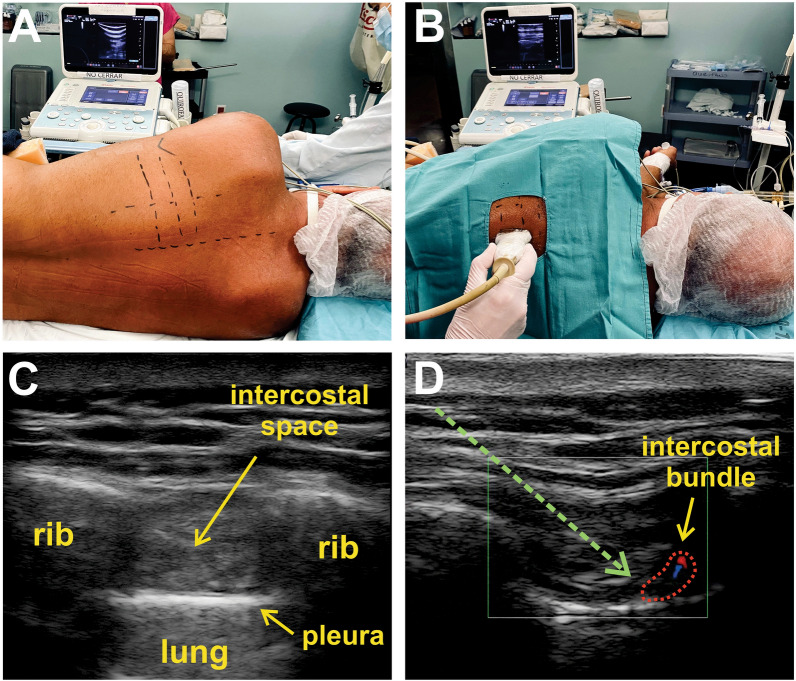
Patient position and standard US anatomical landmarks. The images belong to a 51-year-old male undergoing left lower lobectomy by u-VATS. **A** 4th, 5th, and 6th thoracic intercostal spaces are marked. **B** At those intercostal spaces, an US high-frequency probe is longitudinally placed 7–10 cm parallel to the spine. **C** Sonoanatomy of intercostal space is easily recognized. **D** Color Doppler identifies intercostal vessels and nerve. The dotted green arrow depicted the right direction and tip position of the 14G angiocath used during the procedure

Images from five anesthetized patients undergoing u-VATS are used as examples for this presentation. The local IRB approved this material and the corresponding written consent was obtained from patients. The u-VATS lung resections were the main procedure performed at the 5th thoracic intercostal space (ICS), so the cryoanalgesia was done at the 4th, 5th, and 6th ICS of the corresponding hemithorax. The percutaneous cryoanalgesia technique is as follows: first, the target ICS are marked with an indelible pencil and a topically applied chlorhexidine solution is applied (Fig. 1A). At those intercostal spaces, a high-frequency 12 MHz probe (MyLab Gamma, Esaote, Genova, Italy) is longitudinally placed 7–10 cm parallel to the spine (Fig. 1B). The ICS is easily identified between the upper and lower ribs with the pleura and lung at the bottom of the display (Fig. 1C). The intercostal vessels are identified using color Doppler and the nerve is visualized as a typical hypoechoic honeycomb structure in the transverse plane [16]. The puncture is done in-plane with a 14G angiocath, whose tip is placed close to the intercostal nerve (Fig. 1D). The stylet is retired and 1–2 mL of lidocaine 1% is injected through the 14G catheter to stick out the intercostal bundle by hydrodissection. Later on, the cryoprobe (Cryo-S; Metrum Cryoflex, Warsaw, Poland) is placed throughout the 14G catheter and the cryoanalgesia cycle starts at −78 °C for 3 min. This sequence is repeated at each selected ICS.

## Ultrasound signs

The quality of US images depends on ultrasound factors (i.e., probe frequency and settings), patient factors (i.e., body size, obesity, echo window, anatomical variability), and needle design. Many times, these factors make it difficult to identify target tissues or visualize needles and cryoprobe positions using standard US gray-scale images. Thus, a combination of ultrasound signs is used for neurolocation and to confirm the right position of the ice ball close to the intercostal nerve.

First, color Doppler easily detects the position of intercostal vessels. Slight tilting movements and contrast adjustment are useful to localize the intercostal nerve, which is observed as a hypoechoic honeycomb image when the US beam cuts it in the perpendicular plane. In those cases, where the tiny intercostal nerve is not identified, the cryoprobe must be placed beside the intercostal vessels and between the internal and innermost intercostal muscles, at the midpoint of the intercostal space. This probe localization covers almost all intercostal nerve anatomical dispositions, because 73% of the time the nerve runs in the middle of the ICS [11]. Besides, this cryoprobe placement put the ice ball very close to the upper and lower intercostal borders, where the intercostal nerve is located in the other 27% of cases.

**Fig. 2:**
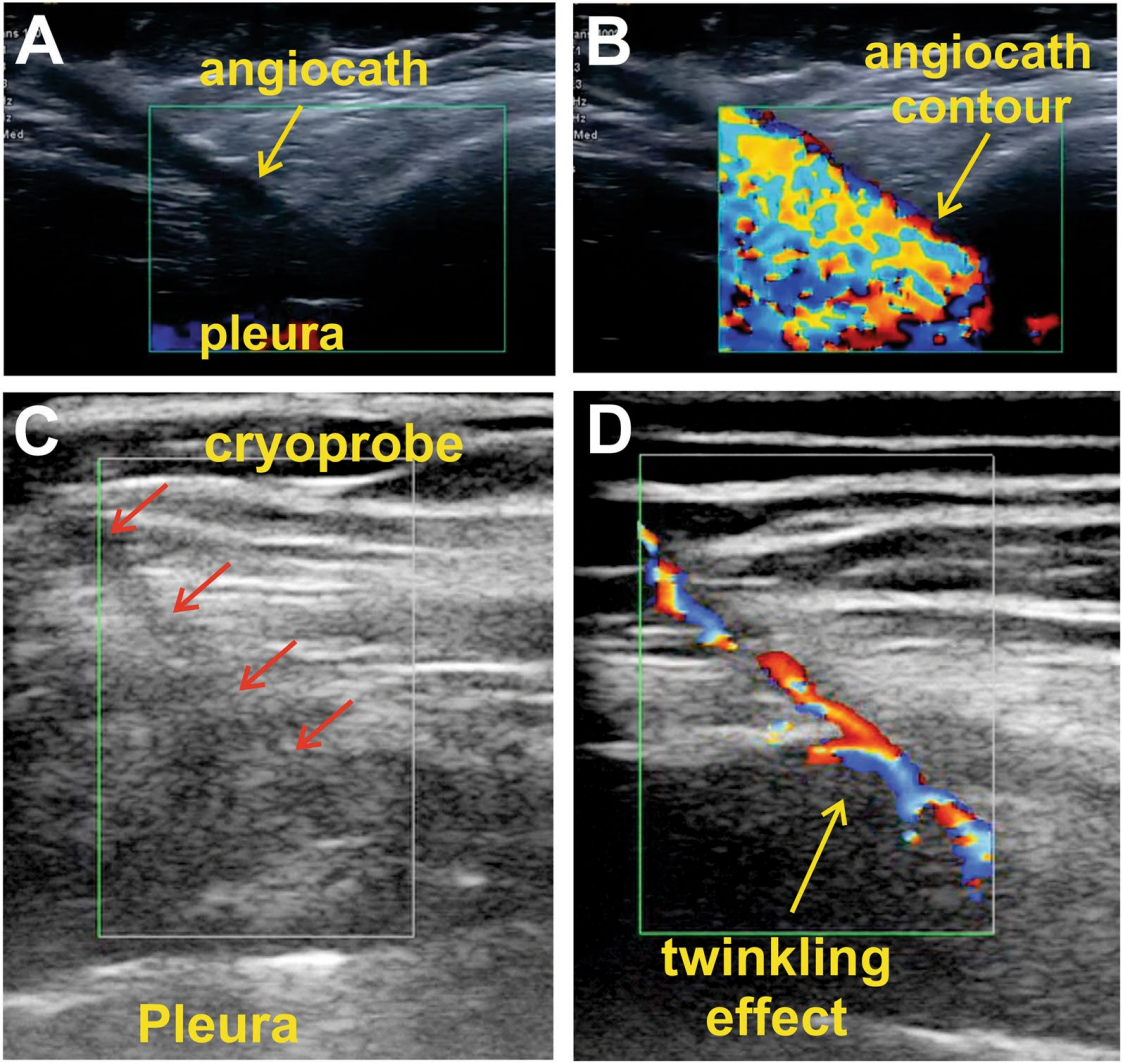
Color Doppler twinkling artifact to visualize the angiocath and cryoprobe positioning. The images belong to a 77-year-old female (Figures **A** and **B**) and a 63-year-old male (Figures **C** and **D**) undergoing lobectomy by u-VATS. **A** Contour of the angiocath catheter (without internal stylet) is visible as a hypoechoic line highlighted by adjusting US contrast. **B** Injection of local anesthetic makes angiocath contour evident by the twinkling artifact. **C** Cryoprobe within the angiocath is not visualized despite tilting and US gray-scale image adjustments (faint shadow marked by red arrows). **D** Twinkling artifact localizing the tip above the pleura

**Fig. 3:**
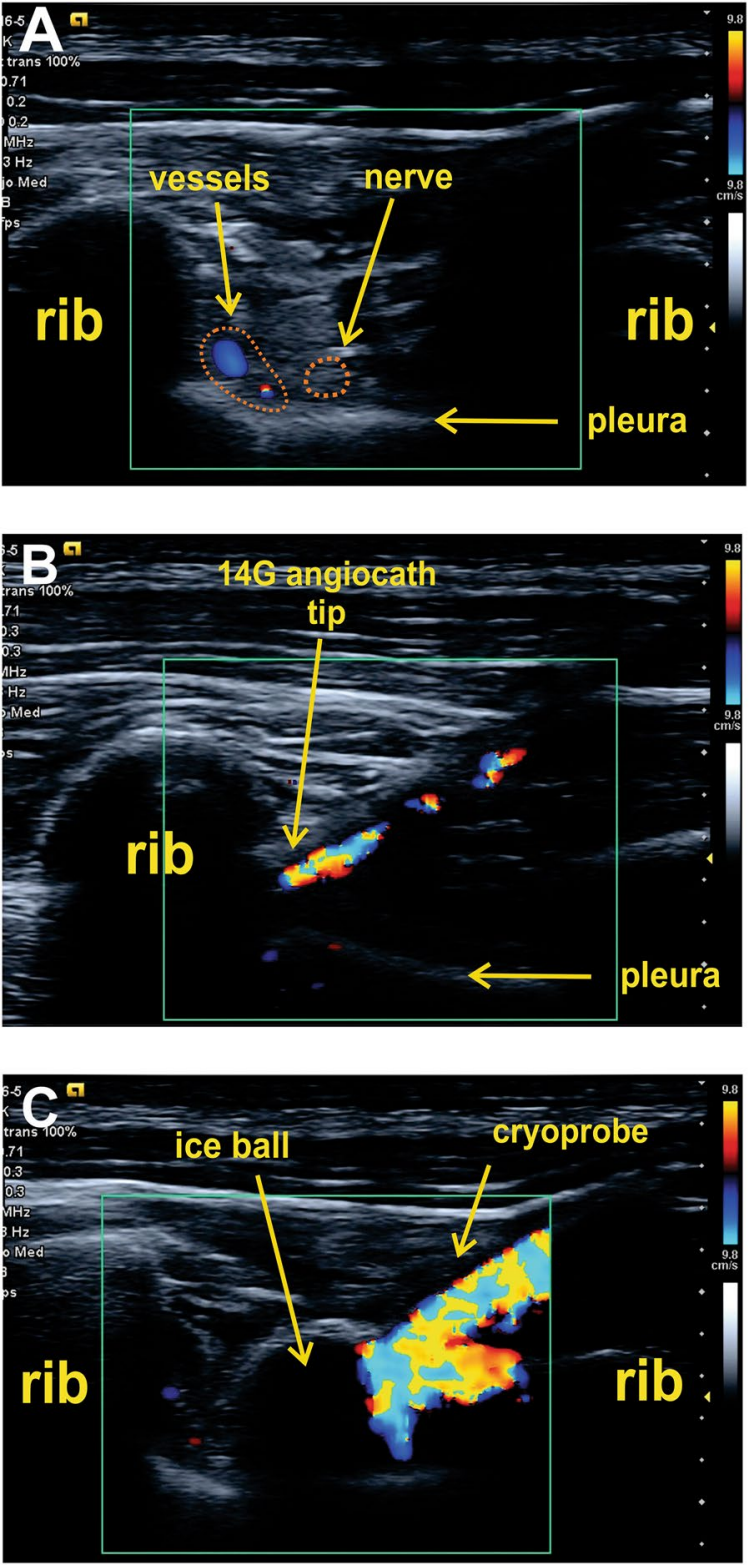
US-guided percutaneous cryoanalgesia sequence. **A** In gray scale images, US contrast is adjusted to magnify anatomical landmarks and the cryoprobe. Then, color Doppler detects the intercostal neurovascular bundle. **B** 14G angiocath is not clearly defined and the twinkling artifacts show that the angiocath tip is placed deep inside the intercostal space beyond the intercostal nerve. **C** Angiocath is then retired a few millimeters until the tip is placed above the nerve. The ice ball is visualized above the intercostal nerve and the cryoprobe contour is highlighted by the twinkling artifact

Many times, the 14G angiocath and cryoprobe are not easily visualized with standard US gray-scale images (Additional file 1: Video S1) [17]. Color Doppler can detect the angiocath and cryoprobe contour with a twinkling artifact [18]. This artifact is generated by a medium composed of multiple rough reflectors, where the acoustic waves split into a complex beam pattern displayed as quickly changing color images that appear below the flat interface [18–21]. The twinkling effect delineates the outer 14G catheter contour during puncture and hydrodissection (Fig. 2A, B and Additional file 2: Video S2) and when the cryoprobe is placed inside the angiocath (Figs. 2C, D and Additional file 3: Video S3). Thus, the twinkling artifact depicts the right position of the cryoprobe tip beside the intercostal nerve and away from the pleural surface (Fig. 3).

Another typical US sign of cryoanalgesia is the ice ball formed at the cryoprobe tip, observed as a hypoechoic spherical image [10]. A pathognomonic feature of the ice ball is the hyperechoic superior rounded border and the inferior acoustic shadowing. This image resembles an ultrasound view of ribs that can confound the operator (Fig. 3 and Additional file 3: Video S3). Sometimes the cryoprobe contour is observed ending in this hypoechoic ice ball, but when the probe is not well defined, color Doppler highlights the cryoprobe by the twinkling artifact (Fig. 3C and Additional file 3: Video S3).

## Commentary

The US is a versatile tool to guide the percutaneous cryoanalgesia in the operating room due to its high availability, portability, non-invasiveness, and ease of use for trained surgical teams. High-frequency US probes help define the tiny intercostal neurovascular bundle. Beyond identification of anatomical landmarks, a critical issue for US-guided regional anesthetic techniques is needle visibility, which depends mainly on needle features. The 14G angiocath is not designed for US-guided procedures and is far away from the ideal needle technical requirements [17]. However, this catheter fits with the cryoprobe size and allows the multi-dose cryoprobe tip to reach the intercostal nerve, preserving its own integrity. Beyond that, the 14G catheter is not specifically designed for US-guided procedures, and its visibility is reduced at steeper insertion angles such as the one needed for the intercostal neural block in adults [22]. This limitation reduces analgesia quality and increases the risk of 14G sharp tip injury to the pleura and intercostal neurovascular bundle [23].

We have described a US technique with a high-frequency probe for guiding precise percutaneous cryoanalgesia. The Doppler twinkling artifact, for example, aids in defining the cryoprobe contour and tip positioning. The visualization of the ice ball at the cryoprobe tip assures the right position for successful intercostal nerve axonotmesis.

The advantages of percutaneous cryoanalgesia over the thoracoscopic approach remain speculative due to the lack of studies on the topic. One main advantage would be the chance to perform percutaneous cryoanalgesia in an ambulatory program days before surgery. This strategy would avoid the long latency of the maximum analgesic effect commonly seen after cryoanalgesia and would decrease the surgical time in the operating room. Surely, new studies are assured to test the feasibility and efficacy of this proposed treatment and its comparison with the thoracic cryoanalgesia technique.

## Conclusions

An ultrasound-guided technique for percutaneous cryoanalgesia of intercostal nerves is described for u-VATS procedures. Ultrasound signs, including the ones observed with color Doppler, help to define anatomical landmarks and to localize the cryoprobe in the right place during the procedure. More clinical studies are necessary to further assess the efficacy and complications of the proposed ultrasound-guided percutaneous cryoanalgesia compared to blind insertion of the cryoprobe into the intercostal space.

## Supplementary Information

**Additional file 1: Video S1.** Figure A depicts the intercostal space (ICS) and its anatomical limits. Video B shows a well-defined cryoprobe that is placed in the right position at the 7^th^ ICS. Contrarily, in Video C, the visualization of the cryoprobe tip is lost when the catheter is placed deep inside the 6th ICS. Video D shows the same example as Video C, but this time the cryoprobe contour is magnified by the twinkling artifact, assuring the right and safe tip positioning during cryoanalgesia.

**Additional file 2: Video S2.** The 14G angiocath is placed in-plane and the stylet is then retired. Lidocaine 1% 1–2 mL was injected through the catheter (hydrodissection) and its contour is easily visualized by the twinkling artifact. The figures on the right hand explain the main findings.

**Additional file 3: Video S3.** The images depict the cryoprobe contour magnified by the twinkling artifact and with the ice ball formed on its tip. A slight tilting movement reveals the sonoanatomy of the intercostal space and highlights the importance of placing the cryoprobe in-plane below the high-frequency US probe. Note that the cryoprobe is invisible outside of the color Doppler area. The ice ball is characterized by a hypoechoic round image with an upper hyperechoic border that mimics a rib. Figures on the right hand depict anatomical landmarks and the most important US signs observed during cryoanalgesia.

## Data Availability

On request.
